# Antarctic Lichens under Long-Term Passive Warming: Species-Specific Photochemical Responses to Desiccation and Heat Shock Treatments

**DOI:** 10.3390/plants11192463

**Published:** 2022-09-21

**Authors:** Catalina Marín, Miloš Barták, Götz Palfner, Pablo Vergara-Barros, Francisco Fernandoy, Josef Hájek, Angélica Casanova-Katny

**Affiliations:** 1 Laboratory of Mycology and Mycorrhiza, Faculty of Natural Sciences and Oceanography, Campus Concepción, Concepción University, Concepción 4030000, Chile; 2 Department of Experimental Biology, Faculty of Science, Masaryk University, Kamenice 5, Building A13/119, 625 00 Brno, Czech Republic; 3 Department of Molecular Genetics and Microbiology, Biological Sciences Faculty, Pontifical Catholic University of Chile, Santiago 8331150, Chile; 4 Isotope Analysis Laboratory, Andrés Bello University, Viña del Mar 2531015, Chile; 5 Laboratory of Plant Ecophysiology, Faculty of Natural Resources, Campus Luis Rivas del Canto, Catholic University of Temuco, Rudecindo Ortega #03694, Temuco 4780000, Chile

**Keywords:** chlorophyll fluorescence, nitrogen isotope, climate change, thermal shock

## Abstract

Climate warming in the Antarctic tundra will affect locally dominant cryptogams. Being adapted to low temperatures and freezing, little is known about the response of the polar lichens’ primary photochemistry to warming and desiccation. Since 2008, we have monitored the ecophysiological responses of lichens to the future warming scenario during a long-term warming experiment through open top chambers (OTCs) on Fildes Peninsula. We studied the primary photochemical response (potential Fv/Fm and effective efficiency of photosystem II YPSII) of different lichen taxa and morphotypes under desiccation kinetics and heat shock experiments. As lichens grow slowly, to observe changes during warming we methodologically focused on carbon and nitrogen content as well as on the stable isotope ratios. Endemic *Himantormia lugubris* showed the strongest effect of long-term warming on primary photochemistry, where PSII activity occurred at a lower %RWC inside the OTCs, in addition to higher Fv/Fm values at 30 °C in the heat shock kinetic treatment. In contrast, *Usnea aurantiaco-atra* did not show any effect of long-term warming but was active at a thallus RWC lower than 10%. Both *Cladonia* species were most affected by water stress, with *Cladonia* aff. *gracilis* showing no significant differences in primary photochemical responses between the warming and the control but a high sensibility to water deficiency, where, at 60% thallus RWC, the photochemical parameters began to decrease. We detected species-specific responses not only to long-term warming, but also to desiccation. On the other hand, the carbon content did not vary significantly among the species or because of the passive warming treatment. Similarly, the nitrogen content showed non-significant variation; however, the C/N ratio was affected, with the strongest C/N decrease in *Cladonia borealis*. Our results suggest that Antarctic lichens can tolerate warming and high temperature better than desiccation and that climate change may affect these species if it is associated with a decrease in water availability.

## 1. Introduction

The latest IPCC report [1] indicates that the effects of climate change on the Antarctic continent differ regionally, with the Antarctic Peninsula experiencing warming very probably since the 1950s, whereas no such trend has been found in East Antarctica. The projected average temperature increase in West Antarctica ranges from 2.3 °C by 2100 under low-emission scenarios (SSP1–2.6) to a 5.6 °C average under a high-emission scenario (SSP5–8.8), in addition to an increase in precipitation such as rain and a decrease in precipitation such as snow. However, warming is not a homogeneous event; it varies regionally and in terms of intensity. In recent years, episodic heatwaves have been registered. Heatwaves are considered extreme events, usually defined as at least three consecutive days with both extreme maximum and minimum temperatures [2] and have rarely been reported for Antarctica. In summer 2020, a three-day heatwave event was registered at Casey Station with a maximum temperature reaching 7.5 °C [2]; similarly, a heatwave recorded in eastern Antarctica marked a 60-year record [3], and the highest temperature of a heatwave so far was registered on the Antarctic Peninsula, at Esperanza Station on Seymour Island, reaching 18.3 °C on 6 February 2020 [4]. González-Herrero et al. [5] indicated that the latest summer heatwaves in Antarctica were at least 0.4 °C warmer than in the previous period, with an overall increase of 25% in magnitude since the period of 1950–1984. The same authors suggest an aggravated severity of these events, which can be largely ascribed to “long-term summer warming of the Antarctic Peninsula rather than recent atmospheric circulation trends”. This heatwave marks a new record and the probability of a new heatwave above 2 °C for 6 days is now about 10 times higher. This raises concerns about the impact of climate change on the cryosphere and the terrestrial and marine ecosystems in Antarctica [6].

The Antarctic Peninsula harbors the highest diversity of terrestrial vegetation on the Antarctic continent [7,8], which must cope with extreme temperatures, high radiation, and strong wind conditions [7,8,9,10,11], as well as freeze–thaw daily cycles. Among the organisms that stand out in these environments are lichens; these are considered stress-tolerant due to their longevity, low nutritional requirements, and specific adaptations [12]. Lichens in Antarctica almost reach the pole and are the most diverse group of organisms, with the highest endemism (30–50%) being higher in continental Antarctica [7,13]. As poikilohydric organisms, they do not possess specialized tissues for water retention and transport: hydration of the thalli occurs when there is rain, fog, or dew and meltwater [14]. Thus, metabolic activity peaks during those periods when hydration of the thalli coincides with low solar radiation conditions and the thallus temperature reaches the range appropriate for photosynthesis [15]. The ecophysiological studies of Antarctic lichens have long focused on the responses of photosynthetic metabolism to low and freezing temperatures [16], showing that the optima for photosynthesis are above 0 °C, but the *Usnea* and *Umbilicaria* species have been shown to be photosynthetically active at −20 °C [17] and at −10 °C, with the photochemical activity measured as Fv/Fm [18].

On the other side, few studies have been dedicated to the response of Antarctic lichens to high temperature. Colesie et al. [19] investigated the acclimatization of the lichens *Stereocaulon alpinum* Laurer, *Usnea aurantiaco-atra* (Jacq.) Bory, and *Placopis contortuplicata* I. M Lamb by exposing them to two levels of high temperature treatments (15 °C and 23 °C) for six weeks, where for all the species an extreme increase in temperature was found to cause the death of the photobionts, while the response to a moderate increase in temperature was variable, with only *S. alpinum* being able to recover its energy homeostasis by increasing the photosynthetic rate, suggesting widely distributed species to be less affected by higher temperature. In [20], work with the endemic species *Himantormia lugubris*, measuring chlorophyll fluorescence parameters and net photosynthetic rate by a gas exchange method at several temperatures (0 °C, 5 °C, 10°, 15 °C, and 20 °C) in fully hydrated thalli, showed that positive net photosynthesis occurred at up to 15 °C.

Lichens in Antarctica are not only exposed to freeze–thaw cycles, but they also undergo a large number of dehydration/rehydration cycles during their lifetime [21]. It has been shown that lichen photosynthetic activity decreases when the dehydration is more severe [22]. According to these authors, lichen desiccation processes correlate with a decline in primary photosynthetic activity, a loss of variable chlorophyll fluorescence (Fv), and a decrease in their overall fluorescence yield. These changes are accompanied by a decoupling of photosystems I and II in the algal/cyanobacterial photobiont during thallus desiccation. The relationship between water content in the thallus and parameters such as Fv/Fm and YPSII has been previously studied in Antarctic lichens, where the observed patterns show species-specific responses [23,24,25]. Barták et al. [23] describe three observable stages when comparing a series of parameters with the RWC: Phase I: supersaturation by high amounts of water; Phase II: an increase/decrease in the fluorescence signal associated with RWC; and Phase III: the drop of the signal in an interval of 30–0% of the RWC (S-type curve). In this study, Phase II and III could be observed but no supersaturation effect was detected, possibly due to the photobiont. The supersaturation effect is found typically in cyanolichens but not in chlorolichens at an RWC of about 100%. The supersaturation effect is characterized by below maximum values for YPSII. Low YPSII is a consequence of suboptimal intrathalline CO_2_ concentration caused by limitation in the diffusion of CO_2_ molecules in fully hydrated cyanolichen thalli. It is believed that exopolysacharidic cellular envelopes in cyanobacteria and their high diffusion resistance for CO_2_ in a fully hydrated state are the underlying mechanism causing the supersaturation effect [26]. Recently, we showed that in several Antarctic lichen species in natural conditions, the dehydration-dependent drop in FV/FM and YPSII was species-specific, starting at an RWC range of 22–32%. The critical RWC for YPSII was below 5%. The changes indicated the involvement of protective mechanisms in the chloroplastic apparatus of lichen photobionts at RWCs of below 20% [25]. What are the major impacts on photosynthetic metabolism of lichens exposed to in situ climatic warming, and how do they improve the response of primary photochemistry to higher temperatures?

Casanova-Katny et al. [27] showed through the continuous monitoring of the electron transport rate (ETR) that *Placopsis antartica* D.J. Galloway and R.I.L. Sm. & Quilhot, under long-term exposure for eight years to a slightly higher temperature inside open-top chambers (OTCs), had a lower ETR than those under control conditions. These are contrasting responses because there is a temperature effect indirectly influencing the water availability for poikilohydric lichens, which are commonly saxicolous species.

In addressing the responses of Antarctic lichens to changes in the environmental conditions, it is important to consider the differences between species. Cho et al. [24] showed in an in situ experiment that the effective quantum yield in *Cladonia borealis* is lower than in *Usnea* sp. living at the same site when there is a lack of precipitation. Barták et al., 2021 [25] studied the inhibition of primary photosynthetic processes in desiccating Antarctic lichens. The response of the potential and effective quantum yields to the decrease in the relative water content of the thalli corresponded to the species-specific factors that can be related with their morphology and acclimation to the microclimate of a particular site.

Lichens, as symbiotic organisms which lack roots, must absorb nutrients through their thalli [28]. It has been described that most Antarctic lichens take up nitrogen coming from penguin colonies, which volatilizes as HN3 and is deposited as NH4 [29]; this is because lichens do not have cuticles that impede the passage of nutrients, as occurs in plants [28]. Therefore, the question arises as to whether Antarctic lichens are able to increase their nitrogen content under the climate warming scenario and whether this is also associated with an increase in carbon uptake, as occurs in plants. The value of the C/N ratio is used in plants to indicate an efficient use of N, where at low nitrogen and high carbon content the ratio decreases, showing a positive effect in plants [30]. What happens in lichens with this parameter will depend on several variables, among them environmental ones, that allow carbon and nitrogen fixation in a balanced way [31]. It has been suggested that there is a regulated C/N economy in lichens, where an increase in N requires an increase in C to store N in non-toxic molecules, such as amino acids, considering that at least 80% of nitrogen in lichens is contained in proteins [32], in order to avoid the accumulation in the thallus of free intracellular ammonium and nitrate [31]. In this case, carbon provides the skeletons for amino acid and protein synthesis and the energy for cellular respiration during days when there is no net carbon fixation. This balance is particularly important in Antarctic lichens, where there are very few days in the Antarctic spring and summer when the temperature is close to the optimum for growth and water is sufficient for optimal photosynthesis [33].

On the other hand, both carbon and nitrogen are absorbed fractionally according to their stable isotopes in the different environmental conditions in which the organisms grow. In this sense, these isotopes act as a fingerprint of the origin of the elements or of the environmental conditions. The nitrogen isotope values show differences associated with the source of origin, where positive values above zero imply an animal origin and negative values below zero are from atmospheric nitrogen. There is a great difference between cyanolichens (symbionts with cyanobacteria), in which the values are always below zero, and chlorolichens, where some species tolerate high concentrations of nitrogen of animal origin; these grow in direct association with these sources in Antarctica, presenting positive values of the N isotope [29]. On the other hand, it has been shown that under conditions of water scarcity, the carbon fixation in photosynthesizing organisms can vary, where very negative values would indicate the uptake of the light isotope, and the change would be associated with a low-water environment, so that the carbon isotope decreases. In this sense, this isotope acts as an indicator of water deficit in the lichen thallus. 

The objective of this study is to analyze the long-term experimental passive warming effects on different lichen species by comparing the primary photochemistry of lichen photosynthesis through the measuring of chlorophyll fluorescence parameters during desiccation and thermal shock kinetics experiments, in addition to elemental and isotope content measurements. We hypothesized that species-specific differences in primary photosynthesis would be attributable to lichen thallus functional morphology and in situ performance in response to prevailing water availability during the growing season.

## 2. Results

### 2.1. Microenvironmental Characterization of Study Sites

**Figure 1:**
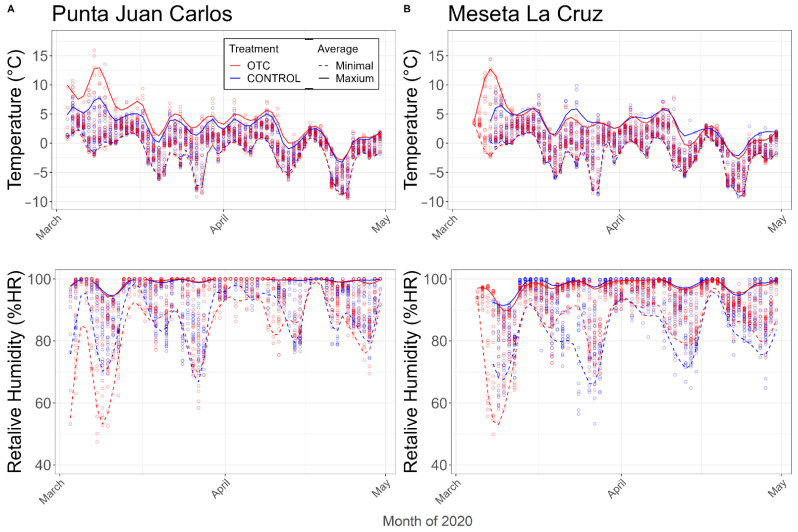
Temperature (°C) and relative air humidity recorded at (**A**) Punta Juan Carlos and (**B**) Meseta La Cruz, Fildes Peninsula, King George Island. Measurements from March to April 2020. The envelope curves are cubic splines fitted through the minima and maxima temperatures of data collected inside (OTC) and outside (control) of open top chambers, and ○ = daily measurements every 2 h.

The same fluctuation patterns in temperature and relative humidity were observed for both sites at Meseta La Cruz and Punta Juan Carlos, for the OTC treatments and the controls (Figure 1). The air temperatures outside and inside the OTCs were close to 0 °C; however, the maximum temperature did show differences between the treatment (OTC) and the control. For Meseta La Cruz, the maximum temperature recorded for the control was 9.8 °C; it was higher in the OTCs, at 14.4 °C. At Punta Juan Carlos, the difference was even greater, with 7.8 °C outside the OTCs (control) and 15.9 °C inside the OTCs (Figure 1). At both sites, inside and outside the OTCs, saturating values of relative humidity (100%) were reached, with an average above 90%. Regarding the minimum humidity recorded at Meseta La Cruz, a low difference of 3.3% was found between the OTCs and the control; in contrast, a high difference was found for the Punta Juan Carlos site, where the minimum level of air humidity recorded inside the OTCs was 10% lower than outside the OTCs (Figure 1).

### 2.2. Desiccation Kinetcs

**Figure 2:**
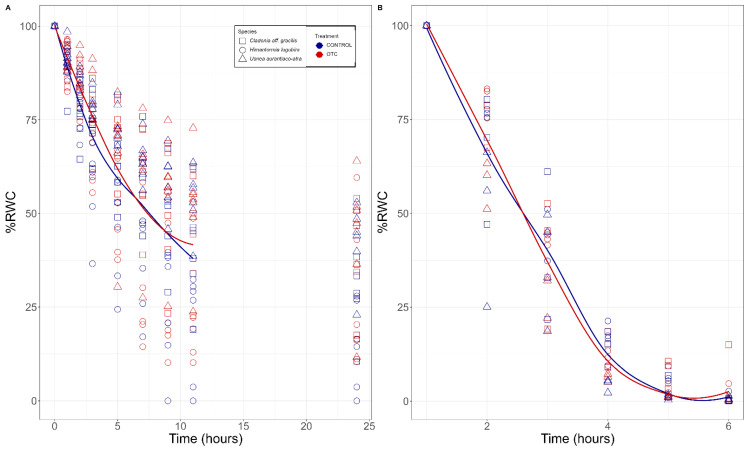
Desiccation kinetics in three different species of Antarctic lichens exposed to passive warming (OTC) and natural conditions (control). (**A**) Lichens %RWC measured at low temperature (4 °C) for 24 h and (**B**) lichens %RWC measured at room temperature, during register of potential and effective quantum yield (Fv/Fm and YPSII).

The desiccation studies were carried out at a low temperature (4 °C) for 24 h in three lichen species, finding the non-significant (*p* < 0.25) lowest values of %RWC in *Himantormia lugubris*, *Cladonia* aff. *gracilis*, and *Usnea aurantiaco-atra*, with 21.3%, 32.9%, and 42.9% respectively, with no significant differences between the OTCs and the control treatments. The %RWC stabilized after 10 h of desiccation (Figure 2A, pooled data of the three species) in samples under low temperature and after six hours at room temperature (Figure 2B).

### 2.3. Chlorophyll Fluorescence vs. Desiccation

**Figure 3:**
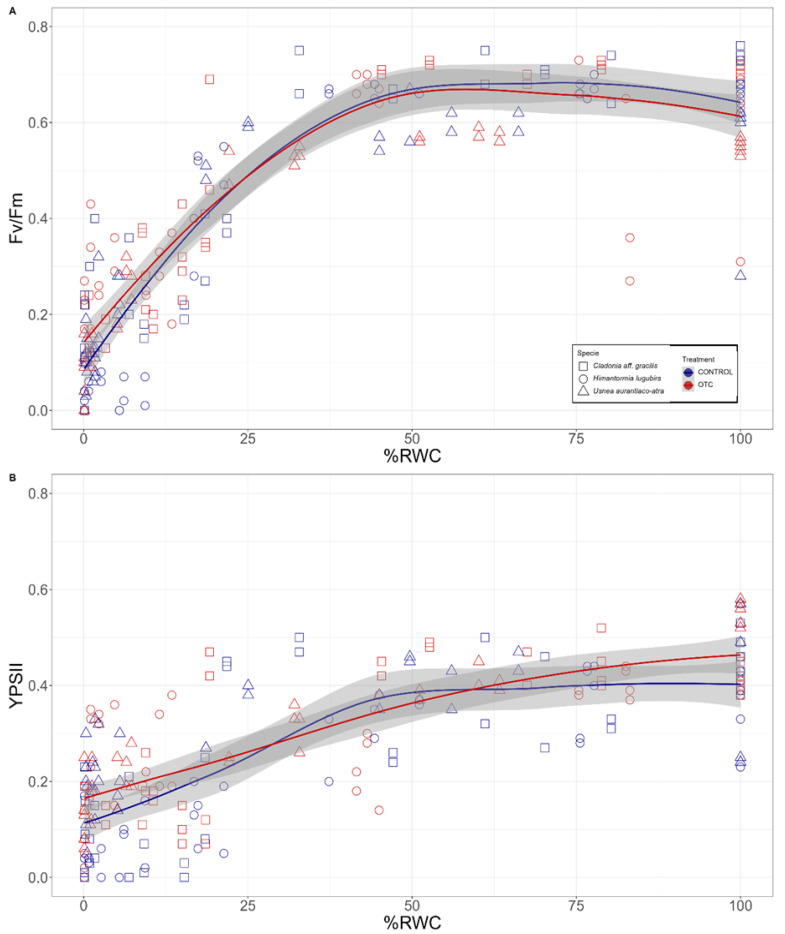
Changes in (**A**) potential photochemical efficiency (Fv/Fm) and (**B**) effective quantum yield (YPSII) in response to desiccation in Antarctic lichens from fully hydrated thalli (RWC = 100%) to a dry state (RWC = 0%). Tendency lines correspond to local polynomial regression fitting, and gray area indicates the standard error of the tendency lines using t-based approximation; n = 6 for each hour and species measured.

For the measurements of chlorophyll florescence kinetics, we used the desiccation kinetics of Figure 2B. The potential photochemical efficiency (Fv/Fm) remained constant for both treatments when the water content declined from 100% to 40%. Within this RWC range, the Fv/Fm values fluctuated between 0.64 ± 0.05 and 0.61 ± 0.11 in the case of the control lichen samples, while, in a similar manner, the OTC samples the Fv/Fm values fluctuated between 0.62 ± 0.11 and 0.60 ± 0.12 (Figure 3A, pooled data of the three species). The effective photochemical efficiency (YPSII) also remained constant for the control samples in the range of 100–40% water content in the thallus, with the values fluctuating between 0.41 ± 0.11 and 0.37 ± 0.08. In the case of the OTC samples, a different pattern of YPSII decrease was observed, registering a maximum of 0.47 ± 0.08 in the fully hydrated thalli and decreasing progressively with dehydration (Figure 3B, pooled data of the three species).

When comparing the values of the Fv/Fm and YPSII in all the desiccation kinetics, no significant differences were found between the OTCs and the control treatments; however, significant differences (*p* < 0.05) were observed when the decrease in the fluorescence began at %RWC values lower than 40% for Fv/Fm and 60% for YPSII, showing that in the control specimens the decrease in the photochemical activity started earlier than in those thalli subjected to passive warming (Table 1). It is worth mentioning that the difference in the RWC values in the thallus when analyzing the linear decrease in the photochemical parameters is due to the differences in the decrease in such parameters, thus ensuring the linear fit of the models.

**Figure 4:**
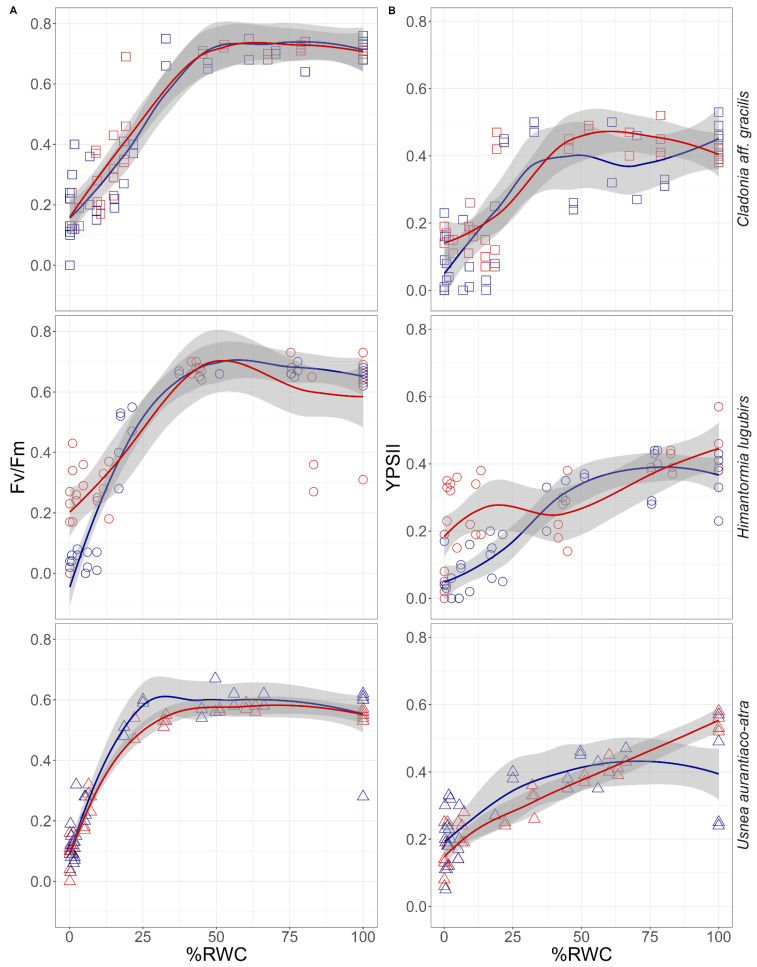
Changes of (**A**) Fv/Fm and (**B**) YPSII upon desiccation, separated by Antarctic lichen species, from a fully hydrated state (RWC = 100%) to a dry state (RWC = 0%). The species correspond to *Cladonia aff. gracilis* (□), *Himantormia lugubris* (○) and *Usnea aurantiaco-atra* (△). Results for samples exposed to passive warming are colored with red and control with blue; tendency lines correspond to local polynomial regression fitting and gray area indicates the standard error of the tendency lines using t-based approximation; n = 6 for each hour measured.

A more detailed analysis by linear fluorescence-decrease models showed species-specific patterns in the chlorophyll fluorescence parameters. In *Cladonia aff. gracilis*, a significant (*p* < 0.05) decrease in the photochemical parameters started at higher thallus water content values (from 60% RWC) than in the other lichens; in *H. lugubris* and *U. aurantiaco-atra*, the decrease of Fv/Fm was linear from 20% RWC and 10%, respectively; although in the case of the YPSII in *U. arantiaco-atra*, this decrease fits the model from 50%, which could mean that this parameter is more sensitive than Fv/Fm to water loss. Finally, when comparing the differences between treatments in each species (*p* < 0.05), for *Cladonia aff. Gracilis* they only differed when comparing the general distribution of the YPSII values; for *H. lugubris*, there were differences in the total comparison in Fv/Fm and for both photochemical parameters when the linear decrease was analyzed, showing that the OTC samples kept a higher fluorescence signal at a lower %RWC when compared with the control samples, but with an earlier decrease. In *U. aurantiaco-atra*, no significant differences between the OTCs and the control were found in any of the analyses performed (Figure 4).

### 2.4. Heat Shock Experiments

**Figure 5:**
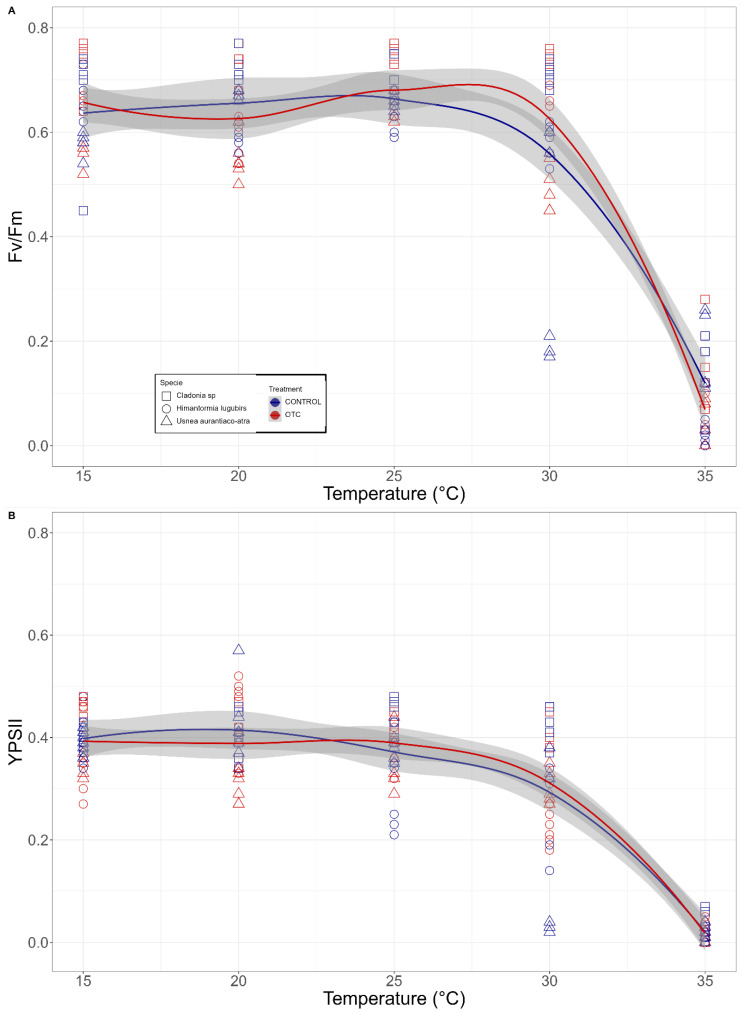
Changes in (**A**) Fv/Fm and (**B**) YPSII in the lichen thalli treated by one-hour thermal shock (15 °C, 20 °C, 25 °C, 30 °C, and 35 °C). Results for samples exposed to passive warming are colored with red and control with blue; tendency lines correspond to local polynomial regression fitting and gray area indicates the standard error of the tendency lines using t-based approximation; n = 6 for each temperature and lichen species.

When analyzing the response of primary photochemistry to the thermal shock treatments without discriminating species, it was observed that, in the case of both potential efficiency and quantum efficiency, the values remained constant between 15 °C and 30 °C. In the above-specified temperature range, the Fv/Fm values fluctuated between 0.66 ± 0.06 and 0.56 ± 0.18 for the control lichens, while for the samples coming from the OTCs the values varied between 0.68 ± 0.05 and 0.63 ± 0.11 in the case of Fv/Fm (Figure 5A, the pooled data of the three species). In the case of YPSII, the values of the control samples varied between 0.41 ± 0.05 and 0.29 ± 0.15, and in the passive warming samples, the values fluctuated between 0.39 ± 0.08 and 0.31 ± 0.08 Fm (Figure 5B, the pooled data of the three species). No significant differences were found between the Fv/Fm and YPSII values obtained in the OTC samples and the outside control samples (Table 1).

**Figure 6:**
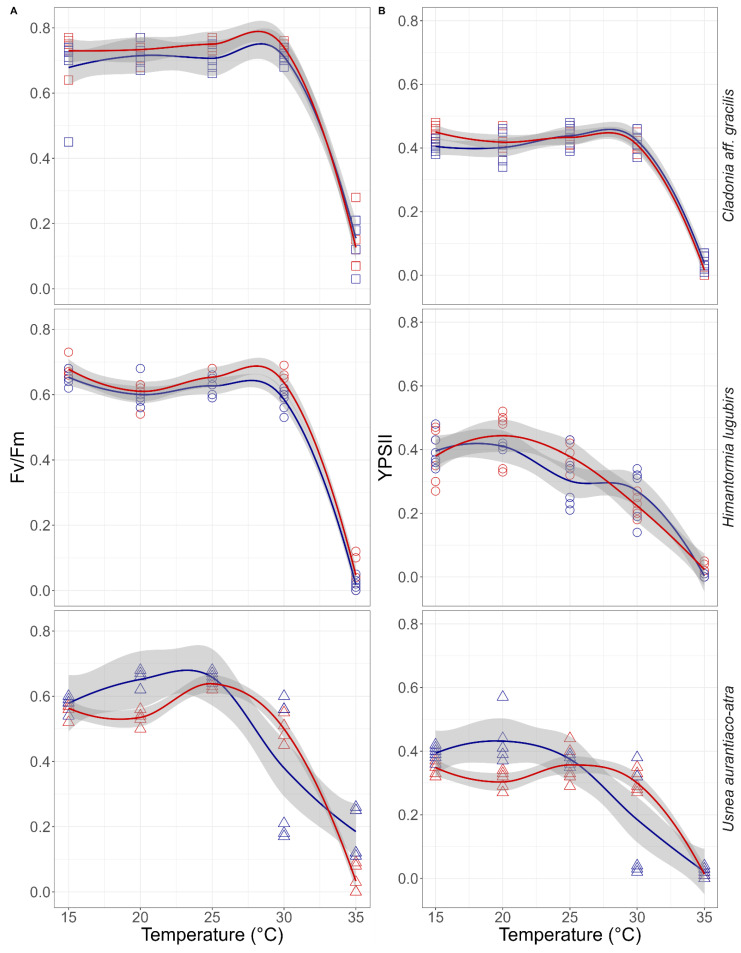
Changes in (**A**) Fv/Fm and (**B**) YPSII upon one-hour thermal shock treatments (for the temperature, see Figure 6 legend) for the Antarctic species *Cladonia aff. gracilis* (□), *Himantormia lugubris*(○), and *Usnea aurantiaco-atra* (△). Results for samples exposed to passive warming are colored with red and control with blue; tendency lines correspond to local polynomial regression fitting, and gray area indicates the standard error of the tendency lines using t-based approximation.

When studying the response of each species to the heat shock experiments, no coherent or constant differences were found between the OTC treatment and the control in *Cladonia aff. gracilis* and *U. aurantiaco-atra*. In *H. lugubris*, however, significant differences (*p* < 0.05) were found when analyzing the drop of the Fv/Fm signal within the temperature interval between 30 and 35 °C, showing that the values in the OTC lichen samples were initially higher than in the control samples (Figure 6 and Table 1).

**Table 1:** Results (*p*-value) of statistical analyses performed to compare chlorophyll fluorescence values (Fv/Fm and YPSII) obtained from three Antarctic lichen species subjected to long-term passive warming treatments through desiccation kinetics experiments and thermal shock treatments; *p*-values are shown for the pool of all species (general) and for individual species for bootstrapping permutations and ANOVA; in the case of one-way ANOVA *p*-values correspond to the section of the curve that fits to a linear theorical model according to Figure 4 and Figure 6.

*p*-Value Bootstrapping					
Experiment		General	*Cladonia aff. gracilis*	*Himantormia lugubris*	*Usnea* *aurantiaco-atra*
Desiccation Kinetics	RWC	0.7	0.8	0.7	0.13
Desiccation vs. photochemical parameters	Fv/Fm	**0.01**	0.16	**0.01**	0.23
	YPSII	0.15	**0.02**	0.23	0.18
Heat shock vs. photochemical parameters	Fv/Fm	0.52	0.63	0.98	0.74
	YPSII	0.61	0.71	0.8	0.05
***p*-Value Linear Model ANOVA**					
Desiccation vs. photochemical parameters	Fv/Fm	**0.03**	0.314	**0.001**	0.46
	YPSII	**0.03**	0.28	**0.001**	0.054
Heat shock vs. photochemical parameters	Fv/Fm	0.78	1.0	**0.01**	0.77
	YPSII	0.69	0.13	0.51	0.16

### 2.5. Elemental and Isotope Comparison

The average relative contents of carbon and nitrogen did not show significant differences between the lichens from the control conditions and those with the OTC treatment. The carbon relative content values were near to 40% in all the samples; the nitrogen relative content varied between species; in *C. borealis*, it was 0.70 ± 0.07% in the control samples and 0.79 ± 0.05% in the samples from the OTCs; in *H. lugubris*, the values were 0.56 ± 0.15% and 0.62 ± 0.16% in the control and the OTC samples, respectively, and finally, for the species *U. aurantiaco-atra*, the average relative content of nitrogen in the control samples was 0.56 ± 0.07% and 0.62 ± 0.08% for the OTCs. (Table 2).

Regarding δ^13^C, *Cladonia borealis* was the only species that had significantly different values between the OTC and control treatments, considering that this species had been exposed to warming for 14 years. The average δ^13^C values in *C. borealis* was −29.0 ± 0.8, and those that were subjected to passive warming had an average value of −27.9 ± 0.3. *H. lugubris* had no significant differences between treatments, with values in the control samples of −25.8 ± 1.3 and −25.1 ± 0.8 in the OTC samples, as in *U. aurantiaco-atra*, which obtained average values of −20.9 ± 0.3 in the control samples and −21.4 ± 2.2 in the samples in the passive warming treatment, with no significant differences between the treatments (Table 2).

Considering the stable isotopic values of nitrogen, *C. borealis* had high positive values of δ^15^N compared with the other species, where the average of the control samples was 6.86 ± 1.31, and in the OTC samples, it was 8.11 ± 3.89, without finding significant differences between the treatments. *H. lugubris* was the only species showing significant differences between the treatments. The average obtained for the control (−8.52 ± 0.85) was lower than the OTC treatments (−6.61 ± 1.45). In *U. aurantiaco-atra*, the values of −10.76 ± 1.85 were obtained in the control samples and −10.14 ± 1.07 in the samples that were subjected to passive warming (Table 2).

The only species that showed differences in C/N between the treatments was *C. borealis*, with the average values obtained in the control samples being higher (57.8 ± 6.3) compared to the results of the OTC samples (50.4 ± 2.4). *H. lugubris* obtained C/N values in the controls which averaged at 74.9 ± 14.8, and in those that were in the OTCs, it was 67.0 ± 17.1. Lastly, for *U. aurantiaco-atra*, the values were 74.5 ± 5.8 in the control samples and 68.1 ± 8.6 in the OTC samples (Table 2).

**Table 2:** Mean content (±standard deviation, n = 3) of stable isotopes (δ15N and δ13C), relative content (%N and %C), and carbon-to-nitrogen ratio (C/N) of different Antarctic lichen species subjected to in situ passive warming (OTC) and under natural conditions (control). Asterisks show significant differences (*p* < 0.05).

Species	Treatment	δ^15^ N	δ^13^ C	%N	%C	C/N
*Cladonia borealis*	Control	6.86 ± 1.31	−29.0 ± 0.8 *	0.70 ± 0.07	40.3 ± 0.2	57.8 ± 6.3 *
	OTC	8.11 ± 3.89	−27.9 ± 0.3 *	0.79 ± 0.05	40.0 ± 0.6	50.4 ± 2.4 *
*Himantormia lugubris*	Control	−8.52 ± 0.85 *	−25.8 ± 1.3	0.56 ± 0.15	40.3 ± 2.0	74.9 ± 14.8
	OTC	−6.61 ± 1.45 *	−25.1 ± 0.8	0.62 ± 0.16	39.4 ± 1.5	67.0 ± 17.1
*Usnea aurantiaco-atra*	Control	−10.76 ± 1.85	−20.9 ± 0.3	0.56 ± 0.07	41.4 ± 0.2	74.5 ± 5.8
	OTC	−10.14 ± 1.07	−21.4 ± 2.2	0.62 ± 0.08	41.8 ± 1.8	68.1 ± 8.6

## 3. Discussion

It has been extensively shown that in the polar environments of the northern hemisphere cryptogams are at a disadvantage compared to vascular plants in the face of rising temperatures, because the latter have higher growth rates and tend to replace the pioneer cryptogams in these environments [34]; however, very few studies refer to the ecophysiology of mosses or lichens themselves in passive warming experiments in situ [35,36,37]. The field studies show that lichens are good bioindicators of climate change effects. The response to changing environmental factors is, however, far from being uniform. The lichens may show increases in their growth rates in the face of rising temperatures or even deleterious effects when the temperatures decrease and snow is maintained for long periods, generating the loss of growth [38].

So far, strong temperature increases associated with climate warming have been observed in Antarctica. Thus, in recent years, there has been an increase in the number of heatwaves, which have become more frequent during the short Antarctic summer, especially in the western area of the Antarctic Peninsula [2]. Bokhorst et al. [39] reported for the first time in Antarctica the loss of 70% of *Usnea antartica* cover after growth under passive warming for 2 and 7 years on Anchorage Island. They suggested that lichens could not store enough metabolic energy during the summer due to high temperatures and were unable to maintain metabolism during the long winter season. However, no measurements of photosynthetic activities were carried out. 

Although Antarctica stores 90% of the water of our planet, it is frozen; so, it is not all available for biological activity [3]. Therefore, water is one of the most important limiting factors in the distribution of organisms in Antarctica [40,41]. Additionally, this is one of the main reasons why Antarctic vegetation is dominated by cryptogams (lichens and bryophytes), and only two vascular plants have been able to permanently establish [42,43]. Due to their poikilohydric characteristics, both mosses and lichens can adapt to the prevailing humidity conditions and rapidly adjust their metabolism. In this context, it is worth asking whether Antarctic lichens can tolerate desiccation and what their photochemical response to this stress is, especially when an increase in air temperature will generate an increase in desiccation due to the rise in evaporation, which occurs at the microenvironment level. In our study, interestingly, the largest differences in primary photochemistry responses were found at low water contents in lichen thalli, where YPSII is a good indicator at RWC values below 60%, and Fv/Fm is more sensitive for detecting changes below 40%.

### 3.1. Primary Photosyntethic Response to Desiccation and Heat Shock Experiments

Our study, comparing the responses of Antarctic lichens exposed to long-term warming, shows that on one hand there are diverse responses to desiccation, especially at very low water content in the thallus. In the correlation between the thallus water contents, Fv/Fm and YPSII showed species-specific responses [23,24,25]. Furthermore, according to the changes observed in Fv/Fm, as well as in YPSII, *Cladonia aff. gracilis* is more sensitive to water loss (with a decrease of YPSII at 60% of the RWC) compared with the other examined species, since it grows in Antarctica and is associated with mosses and, consequently, has a rather abundant and constant water supply. On the other hand, *Usnea aurantiaco-atra*, as a saxicolous species, shows a drop in primary photochemistry at very low values of RWC (Figure 4), without the effect of long-term passive warming, and shows a strong tolerance to desiccation. The only species that shows a significant change due to passive warming is *Himantormia lugubris*, which decreases its photochemical activity below 20% of the RWC, with a more positive response in specimens growing in the OTCs (Figure 4), suggesting an adjustment of the primary photosynthetic processes to a low intrathalline water content under warming. Such behavior, in contrast to the other two species, which showed no difference between the OTCs and the control thalli, may suggest an acclimation potential of *H. lugubris* to maintain relatively high primary photosynthesis even at the RWCs close to the critical point). To support this idea, follow up studies with *H. lugubris* must be conducted in order to analyze the underlying mechanisms, such as, e.g., non-photochemical quenching and its components at an RWC decline from 30 to 0% in OTC-treated lichens. In general, NPQ increased in *H. lugubris* with progressive thallus desiccation at the RWCs under 30% (data not shown), which is similar to the earlier evidence reported for Antarctic lichens (*H. lugubris* [23], *Usnea aurantiaco-atra* [44]). The challenge for the follow-up studies exploiting the chlorophyll fluorescence approach is to evaluate the contribution of the energy dependent, state transition, and photoinhibition-related mechanisms forming a non-photochemical quenching during lichen desiccation. As two morphotypes, and probably ecotypes, of *H. lugubris* that differ in their water supply strategy are reported for King George Island (Antarctica)—Casanova-Katny (unpublished data)—more emphasis should be given to the functional morphology and the likely alternations in photosynthetic performance of the species morphotypes at low RWC. Finally, *U. aurantiaco-atra*, a dominant species in the landscape of the South Shetland Islands, maintains photosystem II functioning with the lowest water values in the thallus, showing no differences between the long-term warming specimens and the control specimens. 

Although we have no information on how the process of warming and drying occurs in the natural environment for these species, previous results have shown a decrease in ETR in *Placopsis antarctica* in passive warming experiments in Antarctica [27]. This drop was maintained longer in specimens inside OTCs compared to the controls, and it was established that low humidity within the OTCs was probably the determining factor for the ETR drop. It has been shown that at high temperatures, most lichens desiccate rapidly and become photosynthetically inactive within a few minutes; so, our study focused on photochemical activity in fully moist thalli at different temperatures. 

*Usnea* species in Antarctica have been previously reported as having photosynthetic rate optima at temperatures close to 15 °C [45], although there are other studies showing that these optima are much lower (0–5 °C), especially when considering not fully hydrated thalli [46]. Our results at a high water content show that photosystem II activity is maintained up to 30 °C, where the maximum values are found between 15–25 °C. In contrast, *Colesie* et al. [19] worked with high-temperature exposure for 6 weeks with different species, demonstrating that positive net photosynthesis is affected in *Usnea* species remaining for long periods under high temperatures without an evident acclimatization process.

An opposite case is the response of *Cladonia aff. gracilis*, where a significant decrease in the fluorescence signal is observed at high RWC values, and the fluorescence values do not change until inhibition at 35 °C. Cho et al. [24], worked with *Cladonia borealis* and *Usnea* spp, describing and comparing the daily patterns of YPSII, showing a decrease when the microenvironmental humidity decreases due to lack of precipitation, a phenomenon less marked in *Usnea*. This requirement of greater water availability is also reflected in their ecological preferences; both *Cladonia borealis* and *Cladonia aff. gracilis* are species that live on mosses, which allow a microenvironment with a higher humidity, unlike the Usnea species, which are saxicolous species in Antarctica. Considering the ecology of the species, and including the fact that this is a cosmopolitan species, which suggests more temperature tolerance than the endemic or native Antarctic species, and given that there are no major differences between the OTC and control specimens, we suggest that for this type of species, water availability could be a more stressful factor than the increase in temperatures; the ecological aspects of their substrate should also be considered. In this context, it has been described that the photosynthetic optimum of some Antarctic moss species occurs at high temperatures above 20 °C [47], suggesting to the authors that widely distributed species are growing under suboptimal conditions in Antarctica. Bednaříková et al. [48], studying three different lichen species, found that *U. antarctica* showed a specific K-band for high temperatures at 35 °C, using an OJIP technique. The PSII of *U. antarctica* showed a high tolerance against thermal shock, as proven by conductivity and chlorophyll fluorescence intensity measurements at high temperatures, suggesting that the PSII of the species was more resistant to high temperature than the other two foliose lichen species used in the above-specified study. Considering our results from the thermal shock experiment (Figure 6), we may say that the Fv/Fm values recorded for *U. aurantiaco-atra* at 30 and 35 °C did not differ from those of *H. lugubris* and *Cladonia aff. gracilis*. Therefore, the three species, if optimally hydrated, would tolerate the climate warming conditions to the same or a similar extent.

Finally, *Himantormia lugubris* has shown to be the species with more differences observed in the samples subjected to passive warming treatment, viz., higher Fv/Fm values at low %RWC values compared to the control, in addition to significant differences before the thermal shock treatments, as we discussed above. *H. lugubris* is an endemic species that has previously been described as vulnerable to environmental changes [20]; therefore, it is expected that there is an effect of the passive warming treatment on the photosynthetic response, in this case allowing the maintaining of primary photochemical activity under water stress as well as higher Fv/Fm values under thermal shock of 30 °C when fully hydrated. It has previously been shown that net positive photosynthesis in this species does not occur above 15 °C in *H. lugubris* thalli [20,49] during long-term high-temperature exposure (six weeks). However, our results showed that photosystem II activity could be maintained up to 30 °C after heat shock of one hour, suggesting that under well-hydrated conditions this species, which frequently grows on mosses or rocks, is adapted to high temperatures for short intervals.

### 3.2. Elemental and Isotopic Comparison

The values of the carbon and nitrogen content were similar to those reported for other Antarctic lichens growing in extreme environment conditions [32,33]; the fluctuating N content was between 0.56 and 0.79% and the carbon content was close to 39–41% for all species. Interestingly, although not significantly, there was a trend of increasing nitrogen content in the lichens growing within the OTCs over time, which may suggest an increase in the metabolic activity of the nitrogen uptake. It should be noted that many studies show the dependence of Antarctic lichens on nitrogen input from penguin colonies via volatile compounds that travel long distances [29]. It is known that lichens use the entire thallus to absorb nitrogen; so, the OTCs would not be an impediment to this occurrence, i.e., a 10–12% increase in nitrogen content may be associated with a positive effect of the warming of this species on nitrogen metabolism, or the increase may be due to a negative effect of dryness inside the OTCs due to the increase in temperatures. In support of this idea, it can only be observed that the δ^13^C values increase in *C. borealis*; so, it would be an indicator of drought within the OTCs. Furthermore, it was found that although the nitrogen content increases, either significantly (*C. borealis*) or not (*Himantormia lugubris* and *Usnea aurantiacoatra*), the C/N ratio shows a contrary trend, with a decrease in this value between 10 and 12%, suggesting that the carbon uptake is not improving inside the OTCs like the nitrogen absorption, which can be an indicator of a toxic accumulation of nitrogen, which could have a negative effect on lichen growth [31,50]. Nevertheless, it is necessary to analyze the content of other compounds (e.g., chitin, chlorophyll), along with the photosynthetic parameters, to have a better understanding of the effects of the OTCs on the carbon/nitrogen balance of these species [51,52,53], and particularly of lichens. As well as *Cladonia aff. gracilis*, as mentioned by Cho et al. [24], *Cladonia borealis* is a species which is sensitive to the decrease in humidity and lives on mosses, probably due its ability to maintain a more humid microenvironment. Under warming, a slight increase in temperatures may lead to faster desiccation in the lichen thalli, which is also associated with the more positive δ^13^C as an indicator of desiccation stress in *Cladonia borealis* inside OTCs [51,54]. The other two species did not indicate changes in δ^13^C due to growth in warming.

With respect to the nitrogen isotopes, we can see three different responses, although only one species shows statistically significant changes. Interestingly, *Himantormia lugubris* shows an OTC effect on the nitrogen isotope, which suggests that the species remains a shorter period in an hydrated and active state; so, the uptake of nitrogen by the liquid state is for a shorter period in the OTCs in contrast to the control samples, which can be reflected in the nitrogen fingerprint [54]. Passive warming had no effect on the isotopic contents in *Usnea autantiaco-atra*, and even though in *Cladonia borealis* it is the only species showing a positive print in δ^15^N in contrast to the negative values reported in the other species analyzed, the decrease in δ^15^N inside the OTCs is not significant and the positive values can be explained because they are growing near a skuas’ nest; hence, there is a constant input of nitrogen of an animal origin [55]. In general, it can be said that the passive warming system has an impact mainly on the nitrogen metabolism, and it is unclear whether this effect is positive or negative; further studies are required to understand in which compounds the absorbed nitrogen is being invested and if it is transformed into increased growth.

## 4. Materials and Methods

### 4.1. Study Site

**Figure 7:**
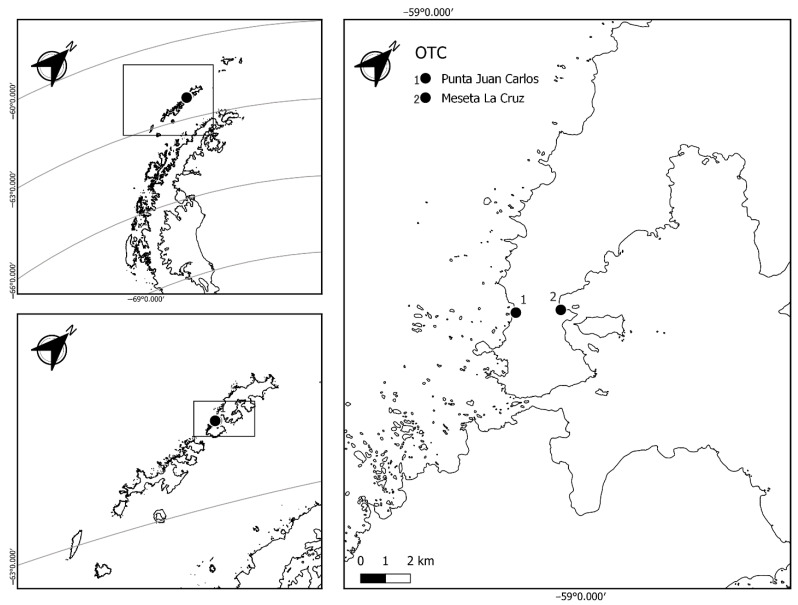
Map of King George Island, South Shetland Islands, west coast of the Antarctic Peninsula. The right panel shows the location of passive warming chambers on the Fildes Peninsula. 1. Punta Juan Carlos; 2. Mesta La Cruz. Map made according to the dataset of Gerrish et al. [59], accessed on 20 April 2020.

**Figure 8:**
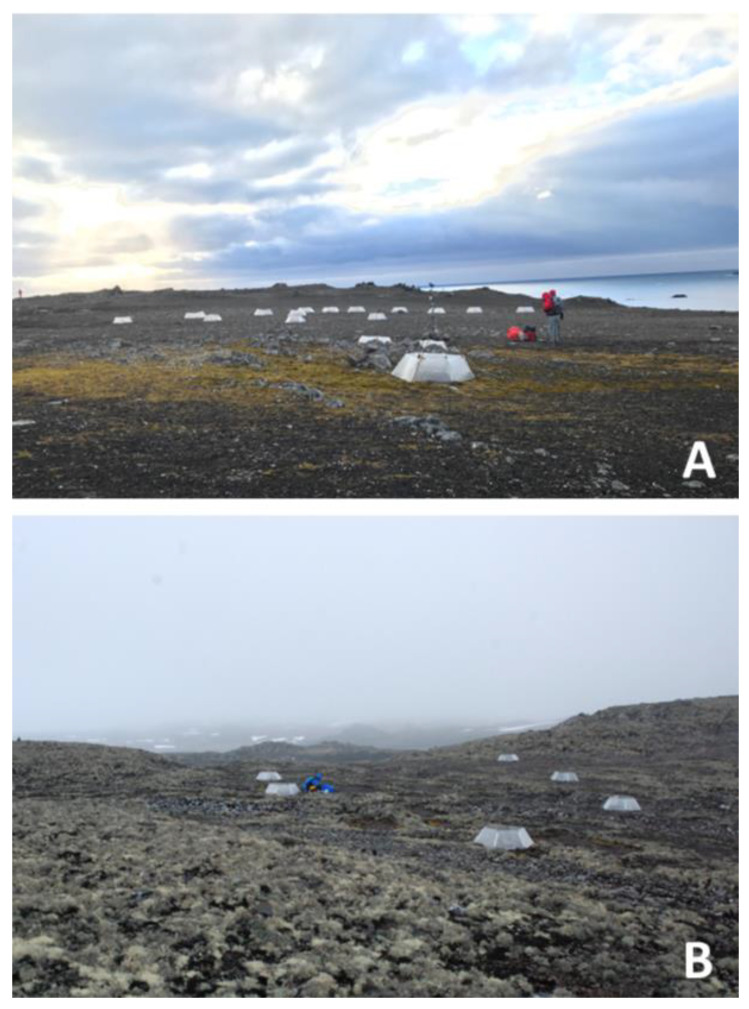
Open-top chambers (OTCs) installed in 2008 in Fildes Peninsula, King George Island, South Shetland Islands, west coast of the Antarctic Peninsula. (**A**) Punta Juan Carlos and (**B**) Meseta La Cruz. Photo credit: A. Casanova-Katny.

The fieldwork was conducted in February 2021 on Fildes Peninsula, King George Island, South Shetland archipelago, at Meseta La Cruz (62°12′ S, 58°57′ W) and Punta Juan Carlos (62°12′ S 58°59′ W) (Figure 7 and Figure 8). Meseta La Cruz is characterized by polygonal soils, with cryptogamic vegetation dominated by lichens; contrastingly, at Punta Juan Carlos, the dominant species is the carpet-forming moss *Sanionia georgicouncinata* (Müll. Hal.) Ochyra, accompanied by the grass *Deschampsia antarctica* E. Desv. The passive warming experiments were carried out using open top chambers (OTCs), which were installed randomly across different vegetation patches and monitored yearly [29,30,51]. The 14-year long-term OTC experiments were installed in 2008 in Meseta la Cruz and in Punta Juan Carlos [56,57] and are composed of 6 transparent acrylic panels 3 mm thick and 40 cm long, joined to form a truncated hexagonal pyramid, according to International Tundra Experiments “ITEX” (https://www.gvsu.edu/itex/, accessed on 9 May 2022); the panels have small perforations to allow better air exchange and avoid excessive heating. To characterize the environmental conditions at both locations, air temperature and relative air humidity sensors (HOBO U23, Onset, Falmouth, Massachusetts, USA) were installed inside and outside the OTCs, recording the values of the variables every three hours from March to May of 2020. In general, our results allow us to estimate that the greatest increase in temperatures occurs during the summer between the OTCs and the control plot, being more marked in the maximum air temperatures [27,35,56,57], where we have recorded increases that fluctuate from 1.4 to 3.3 °C on sunny days [56] at Meseta La Cruz; however, the OTCs at Juan Carlos point to increased mean temperatures of only 0.61 °C. This is in addition to the reports of [58], which show an increase of 0.7 °C in the mean annual temperature of the OTCs with respect to the control. A desirable aspect of working with OTCs is related to snow accumulation, which may be greater within the OTCs; however, on the Fildes Peninsula we have observed that during the Antarctic summer, liquid precipitation increases the most; so, climatic snow events are infrequent. However, it is clear that in both the ex situ studies with controlled climatic chambers and the in situ work with OTCs, there are variables that must be measured continuously to be clear about their effects. In this, case we were able to record temperatures and relative humidity, which indeed decrease at certain times of the day, but then recover.

**Figure 9:**
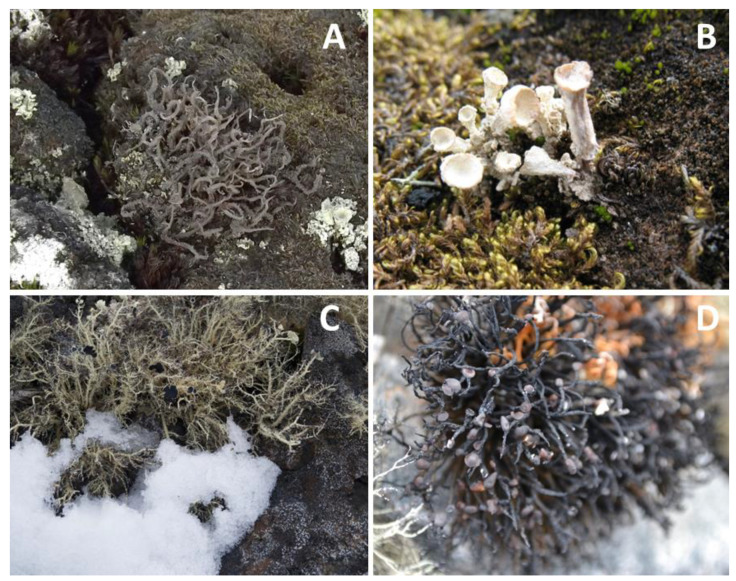
Species used for chlorophyll fluorescence measurement and elemental and isotopic analysis, collected inside and outside of open-top chambers (OTCs), installed in 2008 in Fildes Peninsula, King George Island, South Shetland Islands, west coast of the Antarctic Peninsula. (**A**) *Cladonia aff. gracilis*, (**B**) *Cladonia borealis*, (**C**) *Usnea aurantiaco-atra*, and (**D**) *Himantormia lugubris*. Photo credits: A. Casanova-Katny.

The species used in the study were the chlorolichens *Cladonia borealis* S. Stenroos, *Cladonia aff. gracilis* (L.) Willd, *Himantormia lugubris* (Hue) I.M. Lamb, and *Usnea aurantiaco-atra* (Jacq.) Bory (Table 3, Figure 9).

**Table 3:** Chlorolichen species used in the experimentation with desiccation and thermal shock kinetics, with their respective morphotype, habits, and distribution and the measurements performed.

Family	Species	Morphotype	Habit and Distribution	Measurements
Cladoniaceae	*Cladonia* aff. *gracilis* (L.) Willd.	Squamulose with podetia	On moss and soil. Cosmopolitan	Chlorophyll fluorescence
	*Cladonia borealis* S. Stenroos	Squamulose with podetia	On moss. Antarctica, Asia, Europe, North America, South America	Elemental and isotopic analysis
Parmeliaceae	*Himantormia lugubris* (Hue) I.M. Lamb	Fruticose with flattened branches	On moss and rocks. Endemic to the Antarctic Peninsula.	Chlorophyll fluorescence, Elemental and isotopic analysis
Parmeliaceae	*Usnea aurantiaco-atra* (Jacq.) Bory	Fruticose with cylindrical branches	On rocks. Southern Chile and Argentina to the Antarctic Peninsula.	Chlorophyll fluorescence,
				Elemental and isotopic analysis

After being collected, the samples used for the chlorophyll fluorescence analysis were maintained at a temperature between 3–4 °C at the Laboratory Julio Escudero, Chilean Antarctic Institute (INACH), on Fildes Peninsula. The samples were not stored for more than one day at the time of the experiments mentioned below. In the case of the samples for the elemental and isotopic analysis, these were air dried at 60 °C for 72 h and stored for future transportation.

### 4.2. Desiccation Kinetics

A total of 12 thalli per species (6 from the OTCs and 6 from the natural conditions control) were hydrated with demineralized liquid water with a micropipette until the accumulation of water drops on the thallus was visible; after hydration, the samples were left in individual petri dishes with a wet paper tissue for 24 h to avoid loss of water during the hydration cycle. After the hydration and inspection of the samples (a visual check that paper tissue and the samples were hydrated), the lichen thalli were left to dry at a temperature of 3–4 °C in a refrigerator for 24 h, and the weight of the sample was measured during this period. At the end of the experiment, the samples were dried in an oven at 60 °C for two days, and their dry weight was determined.

The relative water content (RWC) values for each measured time (hours) were obtained from the weight data of each thalli using the equation %RWC = [(Fw − Dw)/(Ww − Dw)] × 100, where Fw corresponds to the weight during the measurement, Dw is the dry weight, and Ww is the weight corresponding to the fully hydrated sample. After the drying experiment for 24 h, a new set of OTC and control samples was selected to analyze the primary photochemical response to the desiccation kinetics.

### 4.3. Determination of Photochemical Efficiency during Desiccation

The response of the primary photosystem II photochemistry during desiccation was analyzed by measuring chlorophyll fluorescence through a FluorCam HFC 1000-H fluorimeter (Photon Systems Instruments, Drásov, Czech Republic) with FluorCam 7.0 software, following the protocol described by Barták et al. [25], obtaining the potential photosystem II yield (Fv/Fm), calculated as Fv/Fm = (Fm − F0/Fm), where Fm corresponds to the maximum chlorophyll fluorescence of dark-adapted chlorophyll, F0 to the minimum fluorescence value of dark-adapted chlorophyll, and Fv corresponds to the variable fluorescence [60]. In parallel with Fv/Fm, the effective photosystem II yield (YPSII) was also measured and calculated as YPSII = (Fm′ − Ft)/Fm′, where Fm’ corresponds to the maximum fluorescence of the light-adapted chlorophyll and Ft corresponds to the steady-state chlorophyll fluorescence in the light-adapted state [54]. The fluorimeter sensitivity was adjusted at 45%, while irradiance was adjusted at 15% for actinic illumination (i.e., 150 µmol m^−2^ s^−1^) and at 70% for a saturating pulse (i.e., 2730 µmol m^−2^ s^−1^). The experimental setup code for Fluorcam 7.0 is available at request.

The desiccation kinetics for this experiment were performed in an interval of six hours, measuring %RWC, Fv/Fm, and YPSII every hour (six measurements in total), for each thalli used (n = 3 from the OTCs and n = 3 for the control), with two replicates of the photochemical parameters measured in each thallus.

### 4.4. Heat Shock Kinetics

Thermal shock experiments were performed with fully hydrated thalli (%RWC = 100, n = 3 for each OTC and control), using a thermoregulated bath where lichens were exposed for one hour at temperatures of 15 °C, 20 °C, 25 °C, 30 °C, and 35 °C in the dark. For each temperature, different thalli were used to avoid acclimation effects or cumulative damage to the photosystems. At the end of the heat exposure, Fv/Fm and YPSII were measured as mentioned above.

### 4.5. Elemental and Isotope Analysis

After the transportation in dry conditions from the Antarctic, the lichen thalli were ground with liquid nitrogen with a mortar until pulverized; the processed samples were sent for chemical analysis to the Isotope Analysis Laboratory, Andrés Bello University, Viña del Mar 2531015, Chile (LAI-UNAB), where the carbon and nitrogen content in the samples was determined, in addition to the stable isotope signatures δ^15^N and δ^13^C. As the standards of L-Glutamic Acid USGS40 and USGS41a, natural and enriched abundances were used, with a total of 3 samples for each treatment of the species.

### 4.6. Statistical Analysis

The statistical analyses were focused on the comparison of the results obtained between the samples from the passive warming and their controls, first by comparing the %RWC for each hour measured in the drying kinetics, then by comparing the primary photochemistry values obtained for six measurements of %RWC, and finally by comparing the photochemical parameters obtained in the OTCs and the control at the different thermal shock temperatures that were recorded.

As the distribution of the data of the total chlorophyll fluorescence parameters response curve did not meet the assumptions for parametric analysis, to analyze the differences between the values obtained in the samples with passive warming treatments and their controls it was decided to perform bootstrapping tests using 9999 Monte Carlo simulations employing the continuity correction suggested by Davinson and Hinkley [61]. Subsequently, to analyze the paired differences in the relative %RWCs between the OTCs and the control, a permutation test was used for the variance of the samples in relative measurements, as well as for each temperature. Finally, after defining the intervals with a pairwise permutations test (data not shown) in both the desiccation and the heat shock experiments where the chlorophyll fluorescence parameters begin to decay, this decrease could fit in theorical linear models; a one-way ANOVA analysis was performed for comparing this section of the response curves

In the case of the elemental and isotopic comparison, the differences in the values obtained from the OTCs compared to the control samples were analyzed using the Kruskal–Wallis test. All analyses were conducted in R version 4.1.2 [62], linear models; one-way ANOVA and the Kruskal–Wallis test were performed with basis software, permutations with the rcompanion package [63] and graphics with the ggplot2 package [64].

## 5. Conclusions

This work shows that the effect of passive warming on the photochemistry of four species of Antarctic lichens is expressed in species-specific response patterns. In wide-spread in sub-Antarctic and maritime Antarctica species, *Usnea aurantiaco-atra*, the exposure to a slight increase in temperatures does not show stress effects in the primary photochemical activity of photosystem II or the carbon and nitrogen content without treatment-dependent differences in either of the measurements performed. For cosmopolitan species, *Cladonia* spp. exhibited a more pronounced negative response, showing less desiccation tolerance in the primary photochemical activity compared with the other species used and more positive δ^13^C in the OTC samples; so, we conclude that a lack in the water resources can be more stressful than only a rise in the temperature in these lichens. In future experiments, the response of the mosses on which *Cladonia* species grow will also be analyzed to have a better understanding of the impact that climate change has on this type of lichens.

For the endemic species, we can evidence more effects of the long-term warming treatments. In *Himantormia lugubris*, the OTC treatment results in higher permanency in the primary photochemical activity in a low %RWC and higher temperature than in the control samples, in addition to a decrease in the nitrogen uptake. This result, in addition to those of other studies, shows that the rise in temperatures may have different responses in distinct steps in the metabolism; hence, it is necessary to use new techniques and experiments to understand the response of this species to the increase in temperatures.

## Data Availability

Not applicable.
